# ASEAN Health in the post-2015 development agenda

**DOI:** 10.1186/1471-2458-14-S1-I2

**Published:** 2014-01-29

**Authors:** Phusit Prakongsai

**Affiliations:** 1 International Health Policy Program (IHPP), Nonthaburi 11000, Thailand

## 

The health sector has led the development success of the MDG era and created an unprecedented opportunity to achieve more health and social development after 2015. The health-related MDGs have raised the profile of global health to the highest political level, mobilized civil society, increased development assistance for health, and contributed to considerable improvements in health outcomes in low- and middle-income countries. Achievements of health-related MDGs among ASEAN countries are remarkable, and it is crucial to keep the momentum for accelerating progress toward the MDG and sustainable development.

Member states of WHO-SEAR proposed that “human well-being and happiness” must be the overarching goal for the post-2015 development agenda. It is recognized that equity and human rights are at the center of development, and well-being is the core contributor for individual and social happiness. The four pillars of well-being and happiness are identified as 1) sustainable and equitable socio-economic development, 2) good and responsible governance, 3) environmental sustainability, and 4) community and cultural participation.

A key component of “well-being and happiness” is universal health coverage which implies equitable access to essential health services with financial and social protection. In addition, to achieve the unfinished MDG agenda and tackling the growing problems of NCDs, a life-course approach comprising continuum of care with balance of the preventive, promotive, curative and palliative aspects of health care has been recommended. Health systems based on the primary health care need to be strengthened to improve efficiency in service delivery, mobilize adequate resources and equitable financing through good governance.

Indicators and targets for each dimension of human well-being must be identified and focused on equity analysis through disaggregated data covering income, age, gender, rural/urban and vulnerable groups. Ownership, intersectoral collaboration, wider involvement of stakeholders, and more partnerships must be leveraged to achieve the post-2015 development agenda.

